# All­yl(ferrocenylmeth­yl)dimethyl­ammonium perchlorate

**DOI:** 10.1107/S1600536812001766

**Published:** 2012-01-21

**Authors:** Ying-Chun Wang

**Affiliations:** a College of Chemistry and Chemical Engineering, Southeast University, Nanjing 210096, People’s Republic of China

## Abstract

The asymmetric unit of the title complex, [Fe(C_5_H_5_)(C_11_H_17_N)]ClO_4_, contains two independent all­yl(ferro­cenyl­meth­yl)dimethyl­ammonium cations and two ClO_4_^−^ anions. The anions are disordered each over two sets of sites, with an occupancy ratio of 0.617 (6):0.383 (6). The distances from the Fe atoms to the centroids of the unsubstituted and substituted cyclo­penta­dienyl (Cp) rings are 1.645 (1)/1.657 (1) and 1.644 (1)/1.647 (1) Å. The dihedral angles between the two Cp rings are 2.49 (3) and 1.45 (4)° in the two ferrocenyl groups of the cations.

## Related literature

For the ferroelectric properties of related amino derivatives, see: Fu *et al.* (2007 ▶, 2008 ▶, 2009 ▶, 2011*a* ▶,*b* ▶,*c* ▶); Fu & Xiong (2008 ▶). For a related compound, see: Chen *et al.* (2010 ▶).
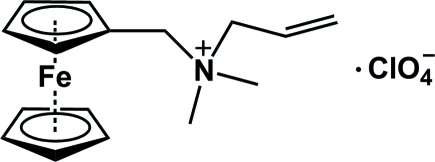


## Experimental

### 

#### Crystal data

[Fe(C_5_H_5_)(C_11_H_17_N)]ClO_4_
*M* *_r_* = 383.65
Monoclinic, 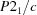

*a* = 15.165 (3) Å
*b* = 10.858 (2) Å
*c* = 25.976 (8) Å
β = 123.84 (2)°
*V* = 3552.7 (17) Å^3^
*Z* = 8
Mo *K*α radiation
μ = 1.02 mm^−1^
*T* = 298 K
0.10 × 0.03 × 0.03 mm

#### Data collection

Rigaku Mercury2 CCD diffractometer
Absorption correction: multi-scan (*CrystalClear*; Rigaku, 2005 ▶) *T*_min_ = 0.910, *T*_max_ = 1.000
35474 measured reflections
8130 independent reflections
4317 reflections with *I* > 2σ(*I*)
*R*_int_ = 0.097

#### Refinement

*R*[*F* ^2^ > 2σ(*F* ^2^)] = 0.065
*wR*(*F* ^2^) = 0.167
*S* = 1.01
8130 reflections
500 parameters
18 restraints
H-atom parameters constrained
Δρ_max_ = 0.38 e Å^−3^
Δρ_min_ = −0.32 e Å^−3^

### 

Data collection: *CrystalClear* (Rigaku, 2005 ▶); cell refinement: *CrystalClear*; data reduction: *CrystalClear*; program(s) used to solve structure: *SHELXS97* (Sheldrick, 2008 ▶); program(s) used to refine structure: *SHELXL97* (Sheldrick, 2008 ▶); molecular graphics: *XP* in *SHELXTL* (Sheldrick, 2008 ▶); software used to prepare material for publication: *SHELXTL*.

## Supplementary Material

Crystal structure: contains datablock(s) I, global. DOI: 10.1107/S1600536812001766/hy2506sup1.cif

Structure factors: contains datablock(s) I. DOI: 10.1107/S1600536812001766/hy2506Isup2.hkl

Additional supplementary materials:  crystallographic information; 3D view; checkCIF report

## Figures and Tables

**Fig. 1.**
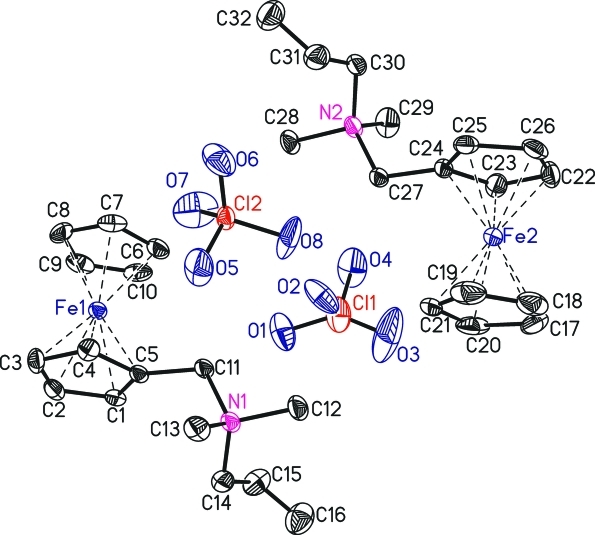
The molecular structure of the title compound. Displacement ellipsoids are drawn at the 30% probability level. H atoms and minor disordered atoms of the perchlorate anions have been omitted for clarity.

## supplementary crystallographic information

### Comment

Simple organic salts containing amino cations have attracted attention as materials, which display ferroelectric-paraelectric phase transitions (Fu *et al.*, 2011*a*, *b*, *c*). With the purpose of obtaining phase transition crystals of amino compounds, various amines have been studied and a series of new materials with this kind of organic molecules have been elaborated (Fu *et al.* 2007, 2008, 2009; Fu & Xiong 2008). Herein we present the crystal structure of the title compound, which can be used as a cation in organic salts.

The asymmetric unit of the title compound contains two independent allyl(ferrocenylmethyl)dimethylammonium cations and two ClO_4_^-^ anions. The anions are disordered each over two sets of sites, with an occupancy ratio of 0.617 (6):0.383 (6) (Fig. 1). The distances from the Fe atoms to the centroids of the unsubstituted and substituted cyclopentadienyl (Cp) rings are 1.645 (1) [1.657 (1) for another ferrocenyl group] and 1.644 (1) [1.647 (1)] Å. The dihedral angles between the two Cp rings are 2.49 (3) and 1.45 (4)° for the two ferrocenyl groups, respectively. The two Cp rings are almost eclipsed with the torsion angles of the two Cp rings (C—Cg1—Cg2—C and C—Cg3—Cg4—C) are in a range of 0.35 (5) to 1.69 (6) and 11.76 (7) to 14.49 (7)°, respectively. All bond lengths and angles are normal and comparable with those in a reported compound (Chen *et al.*, 2010).

### Experimental

A mixture of commercial allyl(ferrocenylmethyl)dimethylamine (0.4 mmol) and HClO_4_ (0.4 mmol) were dissolved in EtOH/distilled water (1:1 *v*/*v*). The solution was slowly evaporated in air, affording red block-shaped crystals of the title compound suitable for X-ray analysis.

The dielectric constant of title compound as a function of temperature indicates that the permittivity is basically temperature-independent, suggesting that this compound should not be a real ferroelectrics or there may be no distinct phase transition occurred within the measured temperature range. Similarly, below the melting point (423 K) of the compound, the dielectric constant as a function of temperature also goes smoothly, and there is no dielectric anomaly observed (dielectric constant ranging from 3.9 to 11.2).

### Refinement

H atoms attached to C atoms were positioned geometrically and treated as riding, with C—H = 0.97 (methylene), 0.98 (ferrocenyl), 0.93 (allyl) and 0.96 (methyl) Å and with *U*_iso_(H) = 1.2(1.5 for methyl)*U*_eq_(C). The ClO_4_^-^ anions are each disordered over two sets of sites, with an occupancy ratio of 0.617 (6):0.383 (6) for both anions.

### Crystal data

**Table:**

[Fe(C_5_H_5_)(C_11_H_17_N)]ClO_4_	*F*(000) = 1600
*M**_r_* = 383.65	*D*_x_ = 1.434 Mg m^−^^3^
Monoclinic, *P*2_1_/*c*	Mo *K*α radiation, λ = 0.71073 Å
Hall symbol: -P 2ybc	Cell parameters from 8130 reflections
*a* = 15.165 (3) Å	θ = 3.0–27.5°
*b* = 10.858 (2) Å	µ = 1.02 mm^−^^1^
*c* = 25.976 (8) Å	*T* = 298 K
β = 123.84 (2)°	Block, red
*V* = 3552.7 (17) Å^3^	0.10 × 0.03 × 0.03 mm
*Z* = 8	

### Data collection

**Table:**

Rigaku Mercury2 CCD diffractometer	8130 independent reflections
Radiation source: fine-focus sealed tube	4317 reflections with *I* > 2σ(*I*)
graphite	*R*_int_ = 0.097
Detector resolution: 13.6612 pixels mm^-1^	θ_max_ = 27.5°, θ_min_ = 3.0°
ω scans	*h* = −19→19
Absorption correction: multi-scan (*CrystalClear*; Rigaku, 2005)	*k* = −14→14
*T*_min_ = 0.910, *T*_max_ = 1.000	*l* = −33→33
35474 measured reflections	

### Refinement

**Table:**

Refinement on *F*^2^	Primary atom site location: structure-invariant direct methods
Least-squares matrix: full	Secondary atom site location: difference Fourier map
*R*[*F*^2^ > 2σ(*F*^2^)] = 0.065	Hydrogen site location: inferred from neighbouring sites
*wR*(*F*^2^) = 0.167	H-atom parameters constrained
*S* = 1.01	*w* = 1/[σ^2^(*F*_o_^2^) + (0.0573*P*)^2^ + 1.691*P*] where *P* = (*F*_o_^2^ + 2*F*_c_^2^)/3
8130 reflections	(Δ/σ)_max_ < 0.001
500 parameters	Δρ_max_ = 0.38 e Å^−^^3^
18 restraints	Δρ_min_ = −0.32 e Å^−^^3^

### Special details

**Table:**

Geometry. All esds (except the esd in the dihedral angle between two l.s. planes) are estimated using the full covariance matrix. The cell esds are taken into account individually in the estimation of esds in distances, angles and torsion angles; correlations between esds in cell parameters are only used when they are defined by crystal symmetry. An approximate (isotropic) treatment of cell esds is used for estimating esds involving l.s. planes.
Refinement. Refinement of F^2^ against ALL reflections. The weighted R-factor wR and goodness of fit S are based on F^2^, conventional R-factors R are based on F, with F set to zero for negative F^2^. The threshold expression of F^2^ > 2sigma(F^2^) is used only for calculating R-factors(gt) etc. and is not relevant to the choice of reflections for refinement. R-factors based on F^2^ are statistically about twice as large as those based on F, and R- factors based on ALL data will be even larger.

### Fractional atomic coordinates and isotropic or equivalent isotropic displacement parameters (Å^2^)

**Table:**

	*x*	*y*	*z*	*U*_iso_*/*U*_eq_	Occ. (<1)
Fe1	0.20107 (4)	0.03124 (5)	0.11383 (3)	0.0524 (2)	
N1	0.3530 (3)	0.4015 (3)	0.15949 (17)	0.0648 (10)	
C1	0.2102 (3)	0.1552 (4)	0.05836 (19)	0.0578 (11)	
H1A	0.1523	0.2079	0.0273	0.069*	
C2	0.2355 (4)	0.0381 (5)	0.0479 (2)	0.0740 (14)	
H2A	0.1974	−0.0060	0.0083	0.089*	
C3	0.3226 (4)	−0.0066 (4)	0.1035 (3)	0.0770 (15)	
H3A	0.3558	−0.0874	0.1094	0.092*	
C4	0.3540 (3)	0.0827 (4)	0.1504 (2)	0.0666 (12)	
H4A	0.4134	0.0759	0.1939	0.080*	
C5	0.2837 (3)	0.1849 (4)	0.12223 (19)	0.0521 (10)	
C6	0.1263 (6)	0.0664 (6)	0.1571 (4)	0.0907 (17)	
H6A	0.1307	0.1437	0.1779	0.109*	
C7	0.1932 (5)	−0.0324 (7)	0.1845 (2)	0.0884 (17)	
H7A	0.2526	−0.0380	0.2280	0.106*	
C8	0.1604 (5)	−0.1253 (5)	0.1394 (4)	0.0961 (18)	
H8A	0.1914	−0.2075	0.1454	0.115*	
C9	0.0727 (5)	−0.0770 (7)	0.0848 (3)	0.097 (2)	
H9A	0.0314	−0.1196	0.0449	0.116*	
C10	0.0531 (4)	0.0401 (7)	0.0968 (3)	0.0938 (19)	
H10A	−0.0043	0.0946	0.0668	0.113*	
C11	0.2828 (3)	0.2957 (4)	0.1554 (2)	0.0610 (11)	
H11A	0.3059	0.2719	0.1971	0.073*	
H11B	0.2104	0.3252	0.1346	0.073*	
C12	0.3459 (4)	0.5026 (5)	0.1963 (3)	0.0912 (17)	
H12A	0.3888	0.5709	0.1995	0.137*	
H12B	0.3709	0.4730	0.2371	0.137*	
H12C	0.2734	0.5288	0.1760	0.137*	
C13	0.4665 (4)	0.3602 (5)	0.1931 (2)	0.0823 (15)	
H13A	0.5101	0.4280	0.1965	0.124*	
H13B	0.4724	0.2944	0.1705	0.124*	
H13C	0.4896	0.3319	0.2338	0.124*	
C14	0.3194 (4)	0.4453 (5)	0.0961 (2)	0.0828 (15)	
H14A	0.3707	0.5052	0.1005	0.099*	
H14B	0.3208	0.3759	0.0730	0.099*	
C15	0.2127 (6)	0.5015 (7)	0.0600 (3)	0.116 (2)	
H15A	0.1554	0.4514	0.0498	0.140*	
C16	0.1935 (7)	0.6173 (7)	0.0427 (3)	0.145 (3)	
H16A	0.2487	0.6704	0.0520	0.174*	
H16B	0.1240	0.6460	0.0206	0.174*	
Fe2	0.31426 (5)	0.66103 (5)	0.38832 (3)	0.0577 (2)	
N2	0.1454 (3)	0.3021 (3)	0.34554 (17)	0.0661 (10)	
C17	0.3271 (7)	0.8145 (6)	0.3495 (5)	0.110 (2)	
H17A	0.2892	0.8922	0.3427	0.131*	
C18	0.4220 (7)	0.7851 (9)	0.3991 (3)	0.109 (2)	
H18A	0.4645	0.8378	0.4356	0.131*	
C19	0.4507 (6)	0.6724 (9)	0.3925 (4)	0.118 (3)	
H19A	0.5174	0.6302	0.4224	0.141*	
C20	0.2927 (6)	0.7162 (9)	0.3082 (3)	0.111 (2)	
H20A	0.2264	0.7120	0.2670	0.133*	
C21	0.3704 (9)	0.6271 (6)	0.3356 (5)	0.116 (3)	
H21A	0.3698	0.5476	0.3176	0.140*	
C22	0.2254 (6)	0.6969 (6)	0.4232 (4)	0.103 (2)	
H22A	0.2000	0.7784	0.4256	0.124*	
C23	0.1718 (4)	0.6162 (5)	0.3733 (3)	0.0792 (14)	
H23A	0.1023	0.6309	0.3350	0.095*	
C24	0.2357 (3)	0.5088 (4)	0.3871 (2)	0.0569 (11)	
C25	0.3212 (6)	0.6439 (6)	0.4686 (3)	0.102 (2)	
H25A	0.3746	0.6817	0.5084	0.123*	
C26	0.3300 (4)	0.5263 (5)	0.4473 (2)	0.0731 (13)	
H26A	0.3893	0.4684	0.4698	0.088*	
C27	0.2137 (3)	0.4046 (4)	0.34492 (19)	0.0584 (11)	
H27A	0.2809	0.3694	0.3559	0.070*	
H27B	0.1786	0.4361	0.3030	0.070*	
C28	0.1349 (4)	0.2021 (4)	0.3024 (2)	0.0844 (15)	
H28A	0.0928	0.1360	0.3025	0.127*	
H28B	0.2041	0.1717	0.3160	0.127*	
H28C	0.1010	0.2346	0.2612	0.127*	
C29	0.0382 (4)	0.3502 (5)	0.3243 (3)	0.107 (2)	
H29A	−0.0070	0.2830	0.3195	0.161*	
H29B	0.0082	0.3915	0.2853	0.161*	
H29C	0.0443	0.4071	0.3544	0.161*	
C30	0.1952 (4)	0.2503 (5)	0.4106 (2)	0.0807 (15)	
H30A	0.2062	0.3175	0.4382	0.097*	
H30B	0.1455	0.1934	0.4101	0.097*	
C31	0.2962 (5)	0.1868 (6)	0.4359 (3)	0.0953 (17)	
H31A	0.3522	0.2322	0.4406	0.114*	
C32	0.3117 (6)	0.0703 (6)	0.4520 (3)	0.128 (2)	
H32A	0.2575	0.0219	0.4480	0.154*	
H32B	0.3783	0.0362	0.4677	0.154*	
Cl2	0.4518 (6)	0.2080 (7)	0.3465 (4)	0.0600 (15)	0.617 (6)
Cl1	0.0269 (7)	0.4646 (9)	0.1473 (4)	0.141 (3)	0.617 (6)
O5	0.5299 (16)	0.220 (2)	0.3347 (10)	0.154 (7)	0.617 (6)
O6	0.5069 (7)	0.1523 (16)	0.4056 (4)	0.139 (4)	0.617 (6)
O7	0.3739 (13)	0.1372 (19)	0.3074 (7)	0.191 (8)	0.617 (6)
O8	0.4272 (13)	0.3283 (8)	0.3485 (12)	0.167 (7)	0.617 (6)
O1	0.0203 (8)	0.3776 (10)	0.1042 (4)	0.173 (4)	0.617 (6)
O2	0.1362 (6)	0.4198 (13)	0.1996 (3)	0.153 (4)	0.617 (6)
O3	0.0544 (18)	0.579 (2)	0.166 (2)	0.311 (8)	0.617 (6)
O4	−0.0535 (12)	0.435 (2)	0.1584 (13)	0.192 (6)	0.617 (6)
Cl2'	0.4528 (16)	0.2164 (19)	0.3435 (11)	0.108 (5)*	0.383 (6)
Cl1'	0.0375 (7)	0.4629 (7)	0.1546 (3)	0.0577 (16)*	0.383 (6)
O3'	−0.0217 (13)	0.5417 (16)	0.0974 (5)	0.154 (7)	0.383 (6)
O8'	0.3523 (15)	0.276 (3)	0.2971 (6)	0.145 (7)	0.383 (6)
O6'	0.4711 (15)	0.281 (3)	0.3921 (9)	0.162 (11)	0.383 (6)
O7'	0.408 (3)	0.101 (2)	0.341 (2)	0.225 (19)	0.383 (6)
O4'	−0.014 (2)	0.468 (4)	0.180 (2)	0.192 (6)	0.383 (6)
O5'	0.511 (2)	0.199 (3)	0.3184 (12)	0.121 (11)	0.383 (6)
O1'	0.104 (3)	0.547 (4)	0.178 (3)	0.311 (8)	0.383 (6)
O2'	0.0743 (17)	0.3640 (18)	0.1613 (10)	0.175 (4)	0.383 (6)

### Atomic displacement parameters (Å^2^)

**Table:**

	*U*^11^	*U*^22^	*U*^33^	*U*^12^	*U*^13^	*U*^23^
Fe1	0.0552 (4)	0.0534 (4)	0.0562 (4)	−0.0074 (3)	0.0356 (3)	−0.0046 (3)
N1	0.077 (2)	0.061 (2)	0.079 (3)	−0.0210 (19)	0.057 (2)	−0.023 (2)
C1	0.059 (3)	0.070 (3)	0.054 (3)	−0.009 (2)	0.037 (2)	0.001 (2)
C2	0.085 (4)	0.078 (4)	0.085 (4)	−0.025 (3)	0.063 (3)	−0.029 (3)
C3	0.076 (3)	0.057 (3)	0.122 (5)	0.002 (2)	0.070 (4)	−0.015 (3)
C4	0.049 (2)	0.069 (3)	0.080 (3)	−0.006 (2)	0.035 (2)	−0.001 (3)
C5	0.053 (2)	0.057 (3)	0.059 (3)	−0.0115 (19)	0.039 (2)	−0.009 (2)
C6	0.114 (5)	0.090 (4)	0.121 (5)	−0.018 (4)	0.098 (5)	−0.013 (4)
C7	0.091 (4)	0.112 (5)	0.062 (3)	−0.025 (4)	0.042 (3)	0.013 (3)
C8	0.123 (5)	0.057 (3)	0.146 (6)	−0.009 (3)	0.099 (5)	0.014 (4)
C9	0.099 (5)	0.129 (6)	0.077 (4)	−0.063 (4)	0.058 (4)	−0.034 (4)
C10	0.063 (3)	0.126 (6)	0.107 (5)	0.003 (3)	0.057 (4)	0.042 (4)
C11	0.068 (3)	0.060 (3)	0.077 (3)	−0.018 (2)	0.054 (3)	−0.017 (2)
C12	0.119 (4)	0.077 (3)	0.115 (4)	−0.033 (3)	0.088 (4)	−0.043 (3)
C13	0.066 (3)	0.095 (4)	0.093 (4)	−0.025 (3)	0.048 (3)	−0.019 (3)
C14	0.106 (4)	0.070 (3)	0.085 (4)	−0.014 (3)	0.062 (4)	−0.008 (3)
C15	0.127 (6)	0.091 (5)	0.117 (5)	−0.010 (4)	0.059 (5)	0.008 (4)
C16	0.171 (7)	0.113 (6)	0.117 (6)	0.015 (5)	0.058 (5)	0.013 (5)
Fe2	0.0790 (5)	0.0511 (4)	0.0594 (4)	−0.0075 (3)	0.0487 (4)	−0.0040 (3)
N2	0.076 (2)	0.065 (2)	0.080 (3)	−0.0181 (19)	0.058 (2)	−0.023 (2)
C17	0.157 (7)	0.065 (4)	0.153 (7)	0.006 (4)	0.114 (6)	0.031 (5)
C18	0.118 (6)	0.139 (7)	0.094 (5)	−0.065 (5)	0.073 (5)	−0.030 (5)
C19	0.109 (5)	0.143 (7)	0.149 (8)	0.033 (5)	0.101 (6)	0.056 (6)
C20	0.132 (6)	0.142 (7)	0.057 (4)	−0.030 (5)	0.052 (4)	0.021 (4)
C21	0.228 (9)	0.070 (4)	0.155 (7)	−0.037 (5)	0.171 (7)	−0.021 (5)
C22	0.153 (6)	0.073 (4)	0.153 (6)	−0.025 (4)	0.128 (6)	−0.038 (4)
C23	0.079 (3)	0.069 (3)	0.111 (4)	−0.004 (3)	0.065 (3)	−0.006 (3)
C24	0.069 (3)	0.052 (3)	0.069 (3)	−0.008 (2)	0.050 (3)	−0.008 (2)
C25	0.164 (6)	0.105 (5)	0.073 (4)	−0.072 (5)	0.088 (4)	−0.040 (4)
C26	0.090 (4)	0.076 (3)	0.053 (3)	−0.020 (3)	0.040 (3)	0.003 (2)
C27	0.068 (3)	0.057 (3)	0.067 (3)	−0.011 (2)	0.048 (2)	−0.009 (2)
C28	0.109 (4)	0.074 (3)	0.087 (4)	−0.034 (3)	0.065 (3)	−0.041 (3)
C29	0.070 (3)	0.107 (5)	0.165 (6)	−0.017 (3)	0.078 (4)	−0.027 (4)
C30	0.124 (5)	0.063 (3)	0.090 (4)	−0.025 (3)	0.081 (4)	−0.021 (3)
C31	0.105 (5)	0.088 (4)	0.091 (4)	−0.010 (4)	0.054 (4)	−0.003 (3)
C32	0.159 (6)	0.087 (5)	0.121 (5)	−0.006 (4)	0.068 (5)	−0.003 (4)
Cl2	0.0578 (19)	0.0518 (16)	0.083 (3)	0.0009 (10)	0.0474 (18)	−0.0096 (13)
Cl1	0.085 (3)	0.143 (4)	0.182 (6)	0.034 (3)	0.066 (3)	−0.020 (3)
O5	0.139 (10)	0.120 (9)	0.28 (2)	−0.016 (7)	0.168 (13)	−0.030 (10)
O6	0.143 (8)	0.191 (10)	0.073 (5)	0.042 (8)	0.054 (5)	0.005 (6)
O7	0.175 (10)	0.200 (18)	0.118 (8)	−0.128 (12)	0.032 (8)	−0.051 (9)
O8	0.148 (13)	0.057 (5)	0.33 (2)	0.018 (6)	0.157 (16)	−0.008 (10)
O1	0.170 (8)	0.194 (8)	0.130 (7)	0.023 (6)	0.069 (7)	−0.057 (7)
O2	0.086 (5)	0.290 (13)	0.072 (5)	0.042 (7)	0.036 (4)	−0.015 (6)
O3	0.19 (2)	0.130 (13)	0.61 (3)	0.012 (11)	0.22 (2)	−0.067 (16)
O4	0.106 (12)	0.175 (13)	0.35 (2)	−0.039 (7)	0.159 (15)	−0.067 (10)
O3'	0.172 (14)	0.171 (14)	0.074 (8)	0.100 (12)	0.041 (8)	0.045 (9)
O8'	0.109 (11)	0.23 (2)	0.095 (10)	0.066 (12)	0.057 (9)	0.005 (11)
O6'	0.100 (12)	0.26 (3)	0.092 (11)	−0.018 (16)	0.035 (10)	−0.080 (15)
O7'	0.39 (4)	0.082 (11)	0.41 (5)	−0.058 (18)	0.35 (5)	−0.02 (2)
O4'	0.106 (12)	0.175 (13)	0.35 (2)	−0.037 (8)	0.160 (15)	−0.067 (10)
O5'	0.16 (2)	0.15 (2)	0.123 (11)	0.078 (17)	0.123 (13)	0.053 (13)
O1'	0.19 (2)	0.130 (13)	0.61 (3)	0.012 (11)	0.22 (2)	−0.067 (16)
O2'	0.171 (8)	0.194 (8)	0.133 (7)	0.024 (7)	0.068 (7)	−0.056 (8)

### Geometric parameters (Å, °)

**Table:**

Fe1—C5	2.024 (4)	Fe2—C22	2.036 (5)
Fe1—C9	2.026 (5)	Fe2—C25	2.038 (5)
Fe1—C7	2.026 (5)	N2—C29	1.490 (6)
Fe1—C6	2.029 (5)	N2—C28	1.503 (5)
Fe1—C1	2.032 (4)	N2—C30	1.523 (6)
Fe1—C10	2.034 (5)	N2—C27	1.527 (5)
Fe1—C4	2.035 (4)	C17—C18	1.329 (9)
Fe1—C8	2.042 (5)	C17—C20	1.393 (9)
Fe1—C3	2.046 (4)	C17—H17A	0.9800
Fe1—C2	2.051 (4)	C18—C19	1.343 (9)
N1—C12	1.500 (5)	C18—H18A	0.9800
N1—C13	1.501 (6)	C19—C21	1.379 (10)
N1—C14	1.505 (6)	C19—H19A	0.9800
N1—C11	1.528 (5)	C20—C21	1.377 (9)
C1—C2	1.398 (6)	C20—H20A	0.9800
C1—C5	1.428 (5)	C21—H21A	0.9800
C1—H1A	0.9800	C22—C25	1.388 (8)
C2—C3	1.394 (7)	C22—C23	1.390 (7)
C2—H2A	0.9800	C22—H22A	0.9800
C3—C4	1.415 (6)	C23—C24	1.428 (6)
C3—H3A	0.9800	C23—H23A	0.9800
C4—C5	1.426 (6)	C24—C26	1.428 (6)
C4—H4A	0.9800	C24—C27	1.478 (5)
C5—C11	1.484 (5)	C25—C26	1.428 (7)
C6—C10	1.354 (8)	C25—H25A	0.9800
C6—C7	1.372 (8)	C26—H26A	0.9800
C6—H6A	0.9800	C27—H27A	0.9700
C7—C8	1.410 (7)	C27—H27B	0.9700
C7—H7A	0.9800	C28—H28A	0.9600
C8—C9	1.397 (8)	C28—H28B	0.9600
C8—H8A	0.9800	C28—H28C	0.9600
C9—C10	1.380 (8)	C29—H29A	0.9600
C9—H9A	0.9800	C29—H29B	0.9600
C10—H10A	0.9800	C29—H29C	0.9600
C11—H11A	0.9700	C30—C31	1.460 (7)
C11—H11B	0.9700	C30—H30A	0.9700
C12—H12A	0.9600	C30—H30B	0.9700
C12—H12B	0.9600	C31—C32	1.312 (7)
C12—H12C	0.9600	C31—H31A	0.9301
C13—H13A	0.9600	C32—H32A	0.9301
C13—H13B	0.9600	C32—H32B	0.9299
C13—H13C	0.9600	Cl2—O7	1.296 (13)
C14—C15	1.477 (8)	Cl2—O8	1.367 (12)
C14—H14A	0.9700	Cl2—O5	1.386 (18)
C14—H14B	0.9700	Cl2—O6	1.412 (13)
C15—C16	1.313 (8)	Cl1—O3	1.31 (3)
C15—H15A	0.9299	Cl1—O1	1.426 (11)
C16—H16A	0.9300	Cl1—O4	1.44 (2)
C16—H16B	0.9300	Cl1—O2	1.523 (11)
Fe2—C21	2.009 (14)	Cl2'—O6'	1.33 (2)
Fe2—C20	2.009 (5)	Cl2'—O5'	1.37 (3)
Fe2—C18	2.013 (6)	Cl2'—O7'	1.42 (3)
Fe2—C17	2.013 (5)	Cl2'—O8'	1.47 (2)
Fe2—C19	2.015 (6)	Cl1'—O2'	1.18 (2)
Fe2—C24	2.027 (4)	Cl1'—O1'	1.24 (5)
Fe2—C23	2.029 (5)	Cl1'—O4'	1.28 (4)
Fe2—C26	2.034 (4)	Cl1'—O3'	1.503 (15)
			
C5—Fe1—C9	158.0 (3)	C18—Fe2—C24	166.4 (4)
C5—Fe1—C7	121.9 (2)	C17—Fe2—C24	152.5 (3)
C9—Fe1—C7	67.0 (2)	C19—Fe2—C24	128.9 (3)
C5—Fe1—C6	106.8 (2)	C21—Fe2—C23	129.4 (3)
C9—Fe1—C6	66.3 (2)	C20—Fe2—C23	109.3 (3)
C7—Fe1—C6	39.5 (2)	C18—Fe2—C23	151.9 (4)
C5—Fe1—C1	41.23 (16)	C17—Fe2—C23	119.5 (3)
C9—Fe1—C1	123.5 (2)	C19—Fe2—C23	167.6 (4)
C7—Fe1—C1	158.4 (2)	C24—Fe2—C23	41.21 (17)
C6—Fe1—C1	123.2 (2)	C21—Fe2—C26	117.7 (3)
C5—Fe1—C10	121.8 (2)	C20—Fe2—C26	151.4 (3)
C9—Fe1—C10	39.7 (2)	C18—Fe2—C26	129.1 (3)
C7—Fe1—C10	66.3 (2)	C17—Fe2—C26	165.8 (3)
C6—Fe1—C10	38.9 (2)	C19—Fe2—C26	108.5 (2)
C1—Fe1—C10	108.5 (2)	C24—Fe2—C26	41.17 (17)
C5—Fe1—C4	41.14 (16)	C23—Fe2—C26	68.8 (2)
C9—Fe1—C4	160.4 (3)	C21—Fe2—C22	167.2 (4)
C7—Fe1—C4	107.3 (2)	C20—Fe2—C22	129.6 (4)
C6—Fe1—C4	122.2 (2)	C18—Fe2—C22	119.8 (3)
C1—Fe1—C4	68.86 (18)	C17—Fe2—C22	109.7 (3)
C10—Fe1—C4	157.2 (3)	C19—Fe2—C22	151.5 (4)
C5—Fe1—C8	158.8 (3)	C24—Fe2—C22	68.56 (19)
C9—Fe1—C8	40.2 (2)	C23—Fe2—C22	40.0 (2)
C7—Fe1—C8	40.6 (2)	C26—Fe2—C22	68.4 (2)
C6—Fe1—C8	67.2 (2)	C21—Fe2—C25	151.8 (4)
C1—Fe1—C8	159.2 (3)	C20—Fe2—C25	166.7 (4)
C10—Fe1—C8	67.3 (2)	C18—Fe2—C25	110.1 (2)
C4—Fe1—C8	123.3 (2)	C17—Fe2—C25	128.6 (3)
C5—Fe1—C3	68.33 (17)	C19—Fe2—C25	119.0 (3)
C9—Fe1—C3	125.7 (3)	C24—Fe2—C25	68.71 (19)
C7—Fe1—C3	124.2 (3)	C23—Fe2—C25	67.5 (2)
C6—Fe1—C3	158.8 (3)	C26—Fe2—C25	41.1 (2)
C1—Fe1—C3	67.58 (19)	C22—Fe2—C25	39.8 (2)
C10—Fe1—C3	161.1 (3)	C29—N2—C28	109.6 (4)
C4—Fe1—C3	40.59 (18)	C29—N2—C30	107.8 (4)
C8—Fe1—C3	109.4 (2)	C28—N2—C30	110.0 (4)
C5—Fe1—C2	68.27 (17)	C29—N2—C27	110.3 (4)
C9—Fe1—C2	110.5 (2)	C28—N2—C27	108.0 (3)
C7—Fe1—C2	160.0 (3)	C30—N2—C27	111.1 (3)
C6—Fe1—C2	159.5 (3)	C18—C17—C20	107.6 (6)
C1—Fe1—C2	40.06 (17)	C18—C17—Fe2	70.7 (4)
C10—Fe1—C2	125.4 (2)	C20—C17—Fe2	69.6 (3)
C4—Fe1—C2	68.03 (19)	C18—C17—H17A	126.2
C8—Fe1—C2	124.4 (2)	C20—C17—H17A	126.2
C3—Fe1—C2	39.77 (19)	Fe2—C17—H17A	126.2
C12—N1—C13	108.3 (4)	C17—C18—C19	110.3 (7)
C12—N1—C14	111.5 (4)	C17—C18—Fe2	70.7 (3)
C13—N1—C14	108.0 (4)	C19—C18—Fe2	70.6 (4)
C12—N1—C11	107.5 (3)	C17—C18—H18A	124.8
C13—N1—C11	110.2 (3)	C19—C18—H18A	124.8
C14—N1—C11	111.3 (3)	Fe2—C18—H18A	124.8
C2—C1—C5	108.0 (4)	C18—C19—C21	107.9 (7)
C2—C1—Fe1	70.7 (3)	C18—C19—Fe2	70.5 (4)
C5—C1—Fe1	69.1 (2)	C21—C19—Fe2	69.7 (4)
C2—C1—H1A	126.0	C18—C19—H19A	126.1
C5—C1—H1A	126.0	C21—C19—H19A	126.1
Fe1—C1—H1A	126.0	Fe2—C19—H19A	126.1
C3—C2—C1	108.6 (4)	C21—C20—C17	107.1 (6)
C3—C2—Fe1	69.9 (3)	C21—C20—Fe2	69.9 (3)
C1—C2—Fe1	69.2 (2)	C17—C20—Fe2	69.9 (3)
C3—C2—H2A	125.7	C21—C20—H20A	126.4
C1—C2—H2A	125.7	C17—C20—H20A	126.4
Fe1—C2—H2A	125.7	Fe2—C20—H20A	126.4
C2—C3—C4	108.9 (4)	C20—C21—C19	107.1 (6)
C2—C3—Fe1	70.3 (3)	C20—C21—Fe2	70.0 (7)
C4—C3—Fe1	69.3 (2)	C19—C21—Fe2	70.2 (7)
C2—C3—H3A	125.5	C20—C21—H21A	126.4
C4—C3—H3A	125.5	C19—C21—H21A	126.4
Fe1—C3—H3A	125.5	Fe2—C21—H21A	126.4
C3—C4—C5	107.1 (4)	C25—C22—C23	108.8 (5)
C3—C4—Fe1	70.1 (3)	C25—C22—Fe2	70.1 (3)
C5—C4—Fe1	69.0 (2)	C23—C22—Fe2	69.7 (3)
C3—C4—H4A	126.5	C25—C22—H22A	125.6
C5—C4—H4A	126.5	C23—C22—H22A	125.6
Fe1—C4—H4A	126.5	Fe2—C22—H22A	125.6
C4—C5—C1	107.3 (4)	C22—C23—C24	108.6 (5)
C4—C5—C11	125.3 (4)	C22—C23—Fe2	70.3 (3)
C1—C5—C11	127.1 (4)	C24—C23—Fe2	69.3 (2)
C4—C5—Fe1	69.8 (2)	C22—C23—H23A	125.7
C1—C5—Fe1	69.7 (2)	C24—C23—H23A	125.7
C11—C5—Fe1	121.7 (2)	Fe2—C23—H23A	125.7
C10—C6—C7	109.2 (6)	C23—C24—C26	106.9 (4)
C10—C6—Fe1	70.7 (3)	C23—C24—C27	126.8 (4)
C7—C6—Fe1	70.1 (3)	C26—C24—C27	126.0 (4)
C10—C6—H6A	125.4	C23—C24—Fe2	69.5 (2)
C7—C6—H6A	125.4	C26—C24—Fe2	69.7 (2)
Fe1—C6—H6A	125.4	C27—C24—Fe2	121.6 (3)
C6—C7—C8	108.2 (5)	C22—C25—C26	108.7 (5)
C6—C7—Fe1	70.3 (3)	C22—C25—Fe2	70.0 (3)
C8—C7—Fe1	70.3 (3)	C26—C25—Fe2	69.3 (3)
C6—C7—H7A	125.9	C22—C25—H25A	125.6
C8—C7—H7A	125.9	C26—C25—H25A	125.6
Fe1—C7—H7A	125.9	Fe2—C25—H25A	125.6
C9—C8—C7	105.6 (5)	C25—C26—C24	106.9 (5)
C9—C8—Fe1	69.3 (3)	C25—C26—Fe2	69.6 (3)
C7—C8—Fe1	69.1 (3)	C24—C26—Fe2	69.2 (2)
C9—C8—H8A	127.2	C25—C26—H26A	126.5
C7—C8—H8A	127.2	C24—C26—H26A	126.5
Fe1—C8—H8A	127.2	Fe2—C26—H26A	126.5
C10—C9—C8	108.7 (5)	C24—C27—N2	115.1 (3)
C10—C9—Fe1	70.4 (3)	C24—C27—H27A	108.5
C8—C9—Fe1	70.5 (3)	N2—C27—H27A	108.5
C10—C9—H9A	125.6	C24—C27—H27B	108.5
C8—C9—H9A	125.6	N2—C27—H27B	108.5
Fe1—C9—H9A	125.6	H27A—C27—H27B	107.5
C6—C10—C9	108.3 (6)	N2—C28—H28A	109.5
C6—C10—Fe1	70.3 (3)	N2—C28—H28B	109.5
C9—C10—Fe1	69.8 (3)	H28A—C28—H28B	109.5
C6—C10—H10A	125.8	N2—C28—H28C	109.5
C9—C10—H10A	125.8	H28A—C28—H28C	109.5
Fe1—C10—H10A	125.8	H28B—C28—H28C	109.5
C5—C11—N1	114.8 (3)	N2—C29—H29A	109.5
C5—C11—H11A	108.6	N2—C29—H29B	109.5
N1—C11—H11A	108.6	H29A—C29—H29B	109.5
C5—C11—H11B	108.6	N2—C29—H29C	109.5
N1—C11—H11B	108.6	H29A—C29—H29C	109.5
H11A—C11—H11B	107.6	H29B—C29—H29C	109.5
N1—C12—H12A	109.5	C31—C30—N2	114.9 (4)
N1—C12—H12B	109.5	C31—C30—H30A	108.5
H12A—C12—H12B	109.5	N2—C30—H30A	108.5
N1—C12—H12C	109.5	C31—C30—H30B	108.5
H12A—C12—H12C	109.5	N2—C30—H30B	108.5
H12B—C12—H12C	109.5	H30A—C30—H30B	107.5
N1—C13—H13A	109.5	C32—C31—C30	123.8 (6)
N1—C13—H13B	109.5	C32—C31—H31A	118.7
H13A—C13—H13B	109.5	C30—C31—H31A	117.5
N1—C13—H13C	109.5	C31—C32—H32A	121.2
H13A—C13—H13C	109.5	C31—C32—H32B	118.8
H13B—C13—H13C	109.5	H32A—C32—H32B	120.0
C15—C14—N1	114.1 (4)	O7—Cl2—O8	116.6 (11)
C15—C14—H14A	108.7	O7—Cl2—O5	113.6 (13)
N1—C14—H14A	108.7	O8—Cl2—O5	101.6 (11)
C15—C14—H14B	108.7	O7—Cl2—O6	109.1 (9)
N1—C14—H14B	108.7	O8—Cl2—O6	112.2 (9)
H14A—C14—H14B	107.6	O5—Cl2—O6	102.8 (10)
C16—C15—C14	124.7 (7)	O3—Cl1—O1	141 (2)
C16—C15—H15A	117.9	O3—Cl1—O4	105.6 (16)
C14—C15—H15A	117.4	O1—Cl1—O4	108.4 (11)
C15—C16—H16A	120.6	O3—Cl1—O2	90.6 (14)
C15—C16—H16B	119.4	O1—Cl1—O2	93.1 (7)
H16A—C16—H16B	120.0	O4—Cl1—O2	111.9 (13)
C21—Fe2—C20	40.1 (3)	O6'—Cl2'—O5'	132 (2)
C21—Fe2—C18	66.3 (3)	O6'—Cl2'—O7'	111 (2)
C20—Fe2—C18	66.2 (3)	O5'—Cl2'—O7'	107 (2)
C21—Fe2—C17	67.3 (3)	O6'—Cl2'—O8'	96.7 (17)
C20—Fe2—C17	40.5 (3)	O5'—Cl2'—O8'	108.5 (19)
C18—Fe2—C17	38.6 (3)	O7'—Cl2'—O8'	96 (2)
C21—Fe2—C19	40.1 (3)	O2'—Cl1'—O1'	114.1 (17)
C20—Fe2—C19	66.9 (3)	O2'—Cl1'—O4'	110 (2)
C18—Fe2—C19	38.9 (3)	O1'—Cl1'—O4'	107 (3)
C17—Fe2—C19	66.0 (3)	O2'—Cl1'—O3'	129.6 (12)
C21—Fe2—C24	107.9 (2)	O1'—Cl1'—O3'	86 (3)
C20—Fe2—C24	118.1 (3)	O4'—Cl1'—O3'	107 (2)
			
C5—Fe1—C1—C2	119.0 (4)	C21—Fe2—C17—C18	−80.0 (5)
C9—Fe1—C1—C2	−82.2 (4)	C20—Fe2—C17—C18	−118.1 (6)
C7—Fe1—C1—C2	164.3 (5)	C19—Fe2—C17—C18	−36.2 (4)
C6—Fe1—C1—C2	−163.9 (3)	C24—Fe2—C17—C18	−165.4 (5)
C10—Fe1—C1—C2	−123.6 (4)	C23—Fe2—C17—C18	156.3 (5)
C4—Fe1—C1—C2	80.6 (3)	C26—Fe2—C17—C18	33.7 (12)
C8—Fe1—C1—C2	−49.1 (7)	C22—Fe2—C17—C18	113.4 (5)
C3—Fe1—C1—C2	36.7 (3)	C25—Fe2—C17—C18	72.7 (6)
C9—Fe1—C1—C5	158.8 (3)	C21—Fe2—C17—C20	38.1 (4)
C7—Fe1—C1—C5	45.3 (6)	C18—Fe2—C17—C20	118.1 (6)
C6—Fe1—C1—C5	77.1 (3)	C19—Fe2—C17—C20	81.9 (5)
C10—Fe1—C1—C5	117.5 (3)	C24—Fe2—C17—C20	−47.3 (7)
C4—Fe1—C1—C5	−38.4 (2)	C23—Fe2—C17—C20	−85.6 (5)
C8—Fe1—C1—C5	−168.1 (5)	C26—Fe2—C17—C20	151.8 (9)
C3—Fe1—C1—C5	−82.2 (3)	C22—Fe2—C17—C20	−128.5 (5)
C2—Fe1—C1—C5	−119.0 (4)	C25—Fe2—C17—C20	−169.2 (5)
C5—C1—C2—C3	0.3 (5)	C20—C17—C18—C19	−0.5 (7)
Fe1—C1—C2—C3	−59.0 (3)	Fe2—C17—C18—C19	59.7 (5)
C5—C1—C2—Fe1	59.2 (3)	C20—C17—C18—Fe2	−60.1 (4)
C5—Fe1—C2—C3	81.8 (3)	C21—Fe2—C18—C17	82.8 (5)
C9—Fe1—C2—C3	−121.7 (4)	C20—Fe2—C18—C17	38.8 (4)
C7—Fe1—C2—C3	−42.9 (7)	C19—Fe2—C18—C17	120.9 (7)
C6—Fe1—C2—C3	161.5 (6)	C24—Fe2—C18—C17	150.2 (8)
C1—Fe1—C2—C3	120.2 (4)	C23—Fe2—C18—C17	−47.9 (7)
C10—Fe1—C2—C3	−163.9 (4)	C26—Fe2—C18—C17	−169.9 (4)
C4—Fe1—C2—C3	37.3 (3)	C22—Fe2—C18—C17	−84.6 (5)
C8—Fe1—C2—C3	−78.9 (4)	C25—Fe2—C18—C17	−127.4 (5)
C5—Fe1—C2—C1	−38.4 (3)	C21—Fe2—C18—C19	−38.1 (5)
C9—Fe1—C2—C1	118.1 (4)	C20—Fe2—C18—C19	−82.1 (5)
C7—Fe1—C2—C1	−163.0 (5)	C17—Fe2—C18—C19	−120.9 (7)
C6—Fe1—C2—C1	41.3 (7)	C24—Fe2—C18—C19	29.3 (12)
C10—Fe1—C2—C1	76.0 (4)	C23—Fe2—C18—C19	−168.8 (5)
C4—Fe1—C2—C1	−82.9 (3)	C26—Fe2—C18—C19	69.2 (6)
C8—Fe1—C2—C1	161.0 (3)	C22—Fe2—C18—C19	154.5 (5)
C3—Fe1—C2—C1	−120.2 (4)	C25—Fe2—C18—C19	111.7 (6)
C1—C2—C3—C4	−0.1 (5)	C17—C18—C19—C21	0.2 (7)
Fe1—C2—C3—C4	−58.7 (3)	Fe2—C18—C19—C21	59.9 (4)
C1—C2—C3—Fe1	58.5 (3)	C17—C18—C19—Fe2	−59.7 (4)
C5—Fe1—C3—C2	−81.6 (3)	C21—Fe2—C19—C18	118.6 (6)
C9—Fe1—C3—C2	79.0 (4)	C20—Fe2—C19—C18	80.2 (5)
C7—Fe1—C3—C2	163.7 (3)	C17—Fe2—C19—C18	35.8 (4)
C6—Fe1—C3—C2	−162.1 (6)	C24—Fe2—C19—C18	−171.5 (4)
C1—Fe1—C3—C2	−37.0 (3)	C23—Fe2—C19—C18	154.8 (10)
C10—Fe1—C3—C2	44.5 (8)	C26—Fe2—C19—C18	−130.1 (5)
C4—Fe1—C3—C2	−120.2 (4)	C22—Fe2—C19—C18	−51.5 (8)
C8—Fe1—C3—C2	120.9 (3)	C25—Fe2—C19—C18	−86.5 (5)
C5—Fe1—C3—C4	38.6 (3)	C20—Fe2—C19—C21	−38.4 (4)
C9—Fe1—C3—C4	−160.8 (3)	C18—Fe2—C19—C21	−118.6 (6)
C7—Fe1—C3—C4	−76.1 (4)	C17—Fe2—C19—C21	−82.7 (5)
C6—Fe1—C3—C4	−41.9 (7)	C24—Fe2—C19—C21	69.9 (5)
C1—Fe1—C3—C4	83.2 (3)	C23—Fe2—C19—C21	36.2 (14)
C10—Fe1—C3—C4	164.7 (6)	C26—Fe2—C19—C21	111.3 (5)
C8—Fe1—C3—C4	−118.9 (4)	C22—Fe2—C19—C21	−170.0 (6)
C2—Fe1—C3—C4	120.2 (4)	C25—Fe2—C19—C21	155.0 (5)
C2—C3—C4—C5	−0.1 (5)	C18—C17—C20—C21	0.6 (7)
Fe1—C3—C4—C5	−59.4 (3)	Fe2—C17—C20—C21	−60.3 (4)
C2—C3—C4—Fe1	59.3 (3)	C18—C17—C20—Fe2	60.8 (4)
C5—Fe1—C4—C3	−118.2 (4)	C18—Fe2—C20—C21	81.0 (5)
C9—Fe1—C4—C3	52.9 (7)	C17—Fe2—C20—C21	117.9 (6)
C7—Fe1—C4—C3	122.7 (4)	C19—Fe2—C20—C21	38.3 (4)
C6—Fe1—C4—C3	163.4 (4)	C24—Fe2—C20—C21	−84.7 (5)
C1—Fe1—C4—C3	−79.8 (3)	C23—Fe2—C20—C21	−129.0 (5)
C10—Fe1—C4—C3	−167.3 (5)	C26—Fe2—C20—C21	−48.1 (7)
C8—Fe1—C4—C3	81.1 (4)	C22—Fe2—C20—C21	−169.2 (4)
C2—Fe1—C4—C3	−36.6 (3)	C25—Fe2—C20—C21	157.3 (11)
C9—Fe1—C4—C5	171.1 (5)	C21—Fe2—C20—C17	−117.9 (6)
C7—Fe1—C4—C5	−119.0 (3)	C18—Fe2—C20—C17	−36.9 (4)
C6—Fe1—C4—C5	−78.3 (4)	C19—Fe2—C20—C17	−79.5 (5)
C1—Fe1—C4—C5	38.5 (2)	C24—Fe2—C20—C17	157.4 (4)
C10—Fe1—C4—C5	−49.1 (6)	C23—Fe2—C20—C17	113.1 (5)
C8—Fe1—C4—C5	−160.7 (3)	C26—Fe2—C20—C17	−166.0 (5)
C3—Fe1—C4—C5	118.2 (4)	C22—Fe2—C20—C17	72.9 (5)
C2—Fe1—C4—C5	81.7 (3)	C25—Fe2—C20—C17	39.4 (13)
C3—C4—C5—C1	0.2 (4)	C17—C20—C21—C19	−0.4 (7)
Fe1—C4—C5—C1	−59.9 (3)	Fe2—C20—C21—C19	−60.7 (4)
C3—C4—C5—C11	175.3 (4)	C17—C20—C21—Fe2	60.3 (4)
Fe1—C4—C5—C11	115.2 (4)	C18—C19—C21—C20	0.2 (7)
C3—C4—C5—Fe1	60.1 (3)	Fe2—C19—C21—C20	60.6 (4)
C2—C1—C5—C4	−0.3 (4)	C18—C19—C21—Fe2	−60.4 (4)
Fe1—C1—C5—C4	59.9 (3)	C18—Fe2—C21—C20	−80.6 (5)
C2—C1—C5—C11	−175.2 (4)	C17—Fe2—C21—C20	−38.5 (4)
Fe1—C1—C5—C11	−115.0 (4)	C19—Fe2—C21—C20	−117.6 (6)
C2—C1—C5—Fe1	−60.3 (3)	C24—Fe2—C21—C20	112.6 (5)
C9—Fe1—C5—C4	−172.0 (5)	C23—Fe2—C21—C20	71.8 (5)
C7—Fe1—C5—C4	79.6 (3)	C26—Fe2—C21—C20	156.2 (4)
C6—Fe1—C5—C4	120.1 (3)	C22—Fe2—C21—C20	40.5 (12)
C1—Fe1—C5—C4	−118.3 (3)	C25—Fe2—C21—C20	−169.2 (5)
C10—Fe1—C5—C4	159.8 (3)	C20—Fe2—C21—C19	117.6 (6)
C8—Fe1—C5—C4	50.0 (6)	C18—Fe2—C21—C19	37.1 (4)
C3—Fe1—C5—C4	−38.1 (3)	C17—Fe2—C21—C19	79.1 (5)
C2—Fe1—C5—C4	−81.0 (3)	C24—Fe2—C21—C19	−129.8 (5)
C9—Fe1—C5—C1	−53.7 (6)	C23—Fe2—C21—C19	−170.5 (4)
C7—Fe1—C5—C1	−162.0 (3)	C26—Fe2—C21—C19	−86.1 (5)
C6—Fe1—C5—C1	−121.6 (3)	C22—Fe2—C21—C19	158.2 (10)
C10—Fe1—C5—C1	−81.9 (3)	C25—Fe2—C21—C19	−51.5 (7)
C4—Fe1—C5—C1	118.3 (3)	C21—Fe2—C22—C25	158.6 (10)
C8—Fe1—C5—C1	168.3 (5)	C20—Fe2—C22—C25	−168.5 (4)
C3—Fe1—C5—C1	80.3 (3)	C18—Fe2—C22—C25	−86.1 (5)
C2—Fe1—C5—C1	37.3 (3)	C17—Fe2—C22—C25	−127.3 (5)
C9—Fe1—C5—C11	68.1 (6)	C19—Fe2—C22—C25	−51.6 (7)
C7—Fe1—C5—C11	−40.2 (5)	C24—Fe2—C22—C25	82.1 (4)
C6—Fe1—C5—C11	0.2 (4)	C23—Fe2—C22—C25	119.9 (5)
C1—Fe1—C5—C11	121.8 (5)	C26—Fe2—C22—C25	37.7 (3)
C10—Fe1—C5—C11	40.0 (5)	C21—Fe2—C22—C23	38.6 (13)
C4—Fe1—C5—C11	−119.8 (5)	C20—Fe2—C22—C23	71.5 (5)
C8—Fe1—C5—C11	−69.9 (7)	C18—Fe2—C22—C23	154.0 (4)
C3—Fe1—C5—C11	−157.9 (4)	C17—Fe2—C22—C23	112.8 (5)
C2—Fe1—C5—C11	159.1 (4)	C19—Fe2—C22—C23	−171.5 (5)
C5—Fe1—C6—C10	120.1 (4)	C24—Fe2—C22—C23	−37.9 (3)
C9—Fe1—C6—C10	−37.6 (4)	C26—Fe2—C22—C23	−82.3 (4)
C7—Fe1—C6—C10	−119.7 (5)	C25—Fe2—C22—C23	−119.9 (5)
C1—Fe1—C6—C10	78.0 (4)	C25—C22—C23—C24	−0.6 (6)
C4—Fe1—C6—C10	162.4 (4)	Fe2—C22—C23—C24	58.9 (3)
C8—Fe1—C6—C10	−81.5 (4)	C25—C22—C23—Fe2	−59.4 (4)
C3—Fe1—C6—C10	−166.7 (5)	C21—Fe2—C23—C22	−169.6 (5)
C2—Fe1—C6—C10	47.5 (8)	C20—Fe2—C23—C22	−129.2 (5)
C5—Fe1—C6—C7	−120.1 (4)	C18—Fe2—C23—C22	−53.8 (7)
C9—Fe1—C6—C7	82.1 (4)	C17—Fe2—C23—C22	−85.9 (5)
C1—Fe1—C6—C7	−162.3 (3)	C19—Fe2—C23—C22	160.8 (11)
C10—Fe1—C6—C7	119.7 (5)	C24—Fe2—C23—C22	119.9 (5)
C4—Fe1—C6—C7	−77.8 (4)	C26—Fe2—C23—C22	81.3 (4)
C8—Fe1—C6—C7	38.2 (3)	C25—Fe2—C23—C22	36.9 (4)
C3—Fe1—C6—C7	−47.0 (7)	C21—Fe2—C23—C24	70.5 (5)
C2—Fe1—C6—C7	167.2 (5)	C20—Fe2—C23—C24	110.9 (4)
C10—C6—C7—C8	−0.2 (6)	C18—Fe2—C23—C24	−173.6 (5)
Fe1—C6—C7—C8	−60.4 (4)	C17—Fe2—C23—C24	154.3 (4)
C10—C6—C7—Fe1	60.2 (4)	C19—Fe2—C23—C24	41.0 (13)
C5—Fe1—C7—C6	77.3 (4)	C26—Fe2—C23—C24	−38.6 (3)
C9—Fe1—C7—C6	−80.0 (4)	C22—Fe2—C23—C24	−119.9 (5)
C1—Fe1—C7—C6	43.8 (7)	C25—Fe2—C23—C24	−82.9 (3)
C10—Fe1—C7—C6	−36.6 (4)	C22—C23—C24—C26	0.4 (5)
C4—Fe1—C7—C6	120.0 (4)	Fe2—C23—C24—C26	59.9 (3)
C8—Fe1—C7—C6	−118.7 (5)	C22—C23—C24—C27	−174.2 (4)
C3—Fe1—C7—C6	161.4 (4)	Fe2—C23—C24—C27	−114.8 (4)
C2—Fe1—C7—C6	−166.9 (5)	C22—C23—C24—Fe2	−59.5 (4)
C5—Fe1—C7—C8	−164.0 (3)	C21—Fe2—C24—C23	−130.1 (5)
C9—Fe1—C7—C8	38.7 (4)	C20—Fe2—C24—C23	−87.7 (4)
C6—Fe1—C7—C8	118.7 (5)	C18—Fe2—C24—C23	167.1 (9)
C1—Fe1—C7—C8	162.5 (5)	C17—Fe2—C24—C23	−54.9 (6)
C10—Fe1—C7—C8	82.1 (4)	C19—Fe2—C24—C23	−169.6 (4)
C4—Fe1—C7—C8	−121.3 (4)	C26—Fe2—C24—C23	118.1 (4)
C3—Fe1—C7—C8	−79.9 (4)	C22—Fe2—C24—C23	36.8 (3)
C2—Fe1—C7—C8	−48.2 (7)	C25—Fe2—C24—C23	79.7 (4)
C6—C7—C8—C9	0.4 (6)	C21—Fe2—C24—C26	111.9 (5)
Fe1—C7—C8—C9	−60.0 (3)	C20—Fe2—C24—C26	154.2 (4)
C6—C7—C8—Fe1	60.4 (4)	C18—Fe2—C24—C26	49.1 (10)
C5—Fe1—C8—C9	157.2 (5)	C17—Fe2—C24—C26	−173.0 (5)
C7—Fe1—C8—C9	116.9 (5)	C19—Fe2—C24—C26	72.4 (5)
C6—Fe1—C8—C9	79.6 (4)	C23—Fe2—C24—C26	−118.1 (4)
C1—Fe1—C8—C9	−44.9 (7)	C22—Fe2—C24—C26	−81.3 (3)
C10—Fe1—C8—C9	37.3 (3)	C25—Fe2—C24—C26	−38.4 (3)
C4—Fe1—C8—C9	−165.8 (4)	C21—Fe2—C24—C27	−8.7 (5)
C3—Fe1—C8—C9	−122.8 (4)	C20—Fe2—C24—C27	33.7 (5)
C2—Fe1—C8—C9	−81.1 (4)	C18—Fe2—C24—C27	−71.5 (10)
C5—Fe1—C8—C7	40.2 (7)	C17—Fe2—C24—C27	66.4 (6)
C9—Fe1—C8—C7	−116.9 (5)	C19—Fe2—C24—C27	−48.2 (5)
C6—Fe1—C8—C7	−37.3 (3)	C23—Fe2—C24—C27	121.4 (5)
C1—Fe1—C8—C7	−161.8 (5)	C26—Fe2—C24—C27	−120.6 (5)
C10—Fe1—C8—C7	−79.6 (4)	C22—Fe2—C24—C27	158.1 (5)
C4—Fe1—C8—C7	77.3 (4)	C25—Fe2—C24—C27	−159.0 (5)
C3—Fe1—C8—C7	120.3 (4)	C23—C22—C25—C26	0.5 (6)
C2—Fe1—C8—C7	162.0 (4)	Fe2—C22—C25—C26	−58.7 (3)
C7—C8—C9—C10	−0.4 (6)	C23—C22—C25—Fe2	59.2 (4)
Fe1—C8—C9—C10	−60.4 (4)	C21—Fe2—C25—C22	−170.1 (5)
C7—C8—C9—Fe1	59.9 (3)	C20—Fe2—C25—C22	41.6 (12)
C5—Fe1—C9—C10	−38.8 (7)	C18—Fe2—C25—C22	112.8 (5)
C7—Fe1—C9—C10	80.1 (4)	C17—Fe2—C25—C22	73.5 (5)
C6—Fe1—C9—C10	36.9 (3)	C19—Fe2—C25—C22	154.7 (5)
C1—Fe1—C9—C10	−78.4 (4)	C24—Fe2—C25—C22	−81.6 (4)
C4—Fe1—C9—C10	156.9 (6)	C23—Fe2—C25—C22	−37.1 (3)
C8—Fe1—C9—C10	119.1 (5)	C26—Fe2—C25—C22	−120.1 (5)
C3—Fe1—C9—C10	−163.4 (4)	C21—Fe2—C25—C26	−50.0 (7)
C2—Fe1—C9—C10	−121.3 (4)	C20—Fe2—C25—C26	161.7 (10)
C5—Fe1—C9—C8	−158.0 (5)	C18—Fe2—C25—C26	−127.1 (5)
C7—Fe1—C9—C8	−39.0 (4)	C17—Fe2—C25—C26	−166.4 (4)
C6—Fe1—C9—C8	−82.3 (4)	C19—Fe2—C25—C26	−85.2 (5)
C1—Fe1—C9—C8	162.5 (3)	C24—Fe2—C25—C26	38.5 (3)
C10—Fe1—C9—C8	−119.1 (5)	C23—Fe2—C25—C26	83.0 (3)
C4—Fe1—C9—C8	37.8 (8)	C22—Fe2—C25—C26	120.1 (5)
C3—Fe1—C9—C8	77.5 (4)	C22—C25—C26—C24	−0.2 (6)
C2—Fe1—C9—C8	119.6 (4)	Fe2—C25—C26—C24	−59.4 (3)
C7—C6—C10—C9	−0.1 (6)	C22—C25—C26—Fe2	59.1 (4)
Fe1—C6—C10—C9	59.7 (4)	C23—C24—C26—C25	−0.1 (5)
C7—C6—C10—Fe1	−59.8 (4)	C27—C24—C26—C25	174.6 (4)
C8—C9—C10—C6	0.4 (6)	Fe2—C24—C26—C25	59.7 (3)
Fe1—C9—C10—C6	−60.1 (4)	C23—C24—C26—Fe2	−59.8 (3)
C8—C9—C10—Fe1	60.4 (4)	C27—C24—C26—Fe2	114.9 (4)
C5—Fe1—C10—C6	−76.9 (4)	C21—Fe2—C26—C25	155.9 (5)
C9—Fe1—C10—C6	119.1 (5)	C20—Fe2—C26—C25	−171.3 (6)
C7—Fe1—C10—C6	37.1 (4)	C18—Fe2—C26—C25	74.9 (5)
C1—Fe1—C10—C6	−120.4 (4)	C17—Fe2—C26—C25	48.5 (11)
C4—Fe1—C10—C6	−41.1 (7)	C19—Fe2—C26—C25	113.2 (5)
C8—Fe1—C10—C6	81.4 (4)	C24—Fe2—C26—C25	−118.3 (5)
C3—Fe1—C10—C6	165.1 (6)	C23—Fe2—C26—C25	−79.7 (4)
C2—Fe1—C10—C6	−161.5 (4)	C22—Fe2—C26—C25	−36.6 (3)
C5—Fe1—C10—C9	164.0 (3)	C21—Fe2—C26—C24	−85.9 (4)
C7—Fe1—C10—C9	−82.0 (4)	C20—Fe2—C26—C24	−53.1 (6)
C6—Fe1—C10—C9	−119.1 (5)	C18—Fe2—C26—C24	−166.8 (4)
C1—Fe1—C10—C9	120.5 (4)	C17—Fe2—C26—C24	166.7 (9)
C4—Fe1—C10—C9	−160.2 (5)	C19—Fe2—C26—C24	−128.5 (4)
C8—Fe1—C10—C9	−37.7 (3)	C23—Fe2—C26—C24	38.6 (3)
C3—Fe1—C10—C9	45.9 (8)	C22—Fe2—C26—C24	81.7 (3)
C2—Fe1—C10—C9	79.3 (4)	C25—Fe2—C26—C24	118.3 (5)
C4—C5—C11—N1	91.1 (5)	C23—C24—C27—N2	−87.9 (5)
C1—C5—C11—N1	−94.8 (5)	C26—C24—C27—N2	98.4 (5)
Fe1—C5—C11—N1	177.8 (3)	Fe2—C24—C27—N2	−174.8 (3)
C12—N1—C11—C5	−177.8 (4)	C29—N2—C27—C24	62.5 (5)
C13—N1—C11—C5	−60.0 (5)	C28—N2—C27—C24	−177.7 (4)
C14—N1—C11—C5	59.9 (5)	C30—N2—C27—C24	−57.0 (5)
C12—N1—C14—C15	−54.8 (6)	C29—N2—C30—C31	173.5 (4)
C13—N1—C14—C15	−173.7 (4)	C28—N2—C30—C31	54.0 (5)
C11—N1—C14—C15	65.2 (5)	C27—N2—C30—C31	−65.5 (5)
N1—C14—C15—C16	115.3 (7)	N2—C30—C31—C32	−118.9 (6)
